# Differences in TGF-β1 signaling and clinicopathologic characteristics of histologic subtypes of gastric cancer

**DOI:** 10.1186/s12885-016-2091-x

**Published:** 2016-02-04

**Authors:** Kyung Ho Pak, Dong Hoon Kim, Hyunki Kim, Do Hyung Lee, Jae-Ho Cheong

**Affiliations:** Department of Surgery, Hallym University Medical Center, Hwasung, Korea; Department of Pathology, Hallym University Medical Center, Hwasung, Korea; Department of Medicine, Yonsei University Graduate School, Seoul, Korea; Department of Pathology, Yonsei University College of Medicine, Seoul, Korea; Depatment of Surgery, Yonsei University College of Medicine, 50-1 Yonsei-ro, Seodaemun-gu, 120-752 Seoul Korea; Department of Biochemistry & Molecular Biology, Yonsei University College of Medicine, Seoul, Korea; Brain Korea 21 PLUS Project for Medical Science, Yonsei University College of Medicine, Seoul, Korea; Open NBI Convergence Technology Research Laboratory, Yonsei University College of Medicine, Seoul, Korea

**Keywords:** TGF-β1, Lauren classification, Gastric cancer

## Abstract

**Background:**

Aberrant TGF-β1 signaling is suggested to be involved in gastric carcinogenesis. However, the role of TGF-β1 in intestinal-type [i-GC] and diffuse-type [d-GC] gastric cancer remains largely unknown. In this study, we evaluated the expression of TGF-β1 signaling molecules and compared the clinicopathological features of i-GC and d-GC.

**Methods:**

Patients (n=365, consecutive) who underwent curative gastrectomy for gastric adenocarcinoma in 2005 were enrolled. We performed immunohistochemical staining of TGF-β1, TGF-β1 receptor-2 (TβR2), Smad4, p-ERK1/2, TGF-activated kinase (TAK)1, and p-Akt in 68 paraffin-embedded tumor blocks (33 i-GC and 35 d-GC), scored the expression according to the extent of staining, and evaluated differences between the histologic subtypes.

**Results:**

Patients with d-GC differed from those with i-GC as follows: younger and more likely to be female; more aggressive stage; higher recurrence rate. The expression of TGF-β1 and TβR2 was higher in i-GC (*P* = 0.05 and *P* <0.001, respectively). The expression of Smad4, a representative molecule of the Smad-dependent pathway, was decreased in both subtypes. TAK1 and p-Akt, two major molecules involved in the Smad-independent pathway, were over-expressed (69 ~ 87 % of cases stained), without a statistically significant difference between i-GC and d-GC. Of note, the expression of p-ERK1/2, a Smad-independent pathway, was significantly increased in i-GC (*P* = 0.008).

**Conclusions:**

The clinicopathological characteristics vary in different histologic gastric cancer subtypes. Although TGF-β1 signaling in gastric cancer cells appears hyper-activated in i-GC compared to d-GC, the Smad-dependent pathway seems down-regulated while the Smad-independent pathway seems up-regulated in both histologic subtypes.

**Electronic supplementary material:**

The online version of this article (doi:10.1186/s12885-016-2091-x) contains supplementary material, which is available to authorized users.

## Background

In 2012, gastric cancer was responsible for 723,000 deaths and was ranked as the world’s third leading cause of cancer mortality [1]. Gastric cancer was also the second most common malignancy in Korea [2]. Although conventional chemotherapy has improved the overall prognosis of gastric cancer, the survival rate of patients with advanced cancer still falls short of expectations. With the recent advances in our understanding of the molecular basis of this deadly disease, deregulated cellular pathways were identified and targeted, providing new therapeutic options beyond conventional chemotherapies. Indeed, human epidermal growth factor receptor 2 (HER2) and vascular endothelial growth factor receptor 2 (VEGFR2) have been evaluated as therapeutic targets and are now available as treatment targets in metastatic gastric cancer [3, 4].

Data from one multicenter transcriptome study [5] and The Cancer Genome Atlas [6] established the significance of transforming growth factor beta 1 (TGF)-β1 signaling on gastric cancer progression, supporting its role as an emerging candidate biomarker for gastric cancer. In line with these pivotal studies, others also showed the relation between high expression of TGF-β1 and unfavorable prognosis of gastric cancer patients [7–10].

TGF-β1 has an important role not only in normal physiologic functions like embryonic development, angiogenesis, fibrosis, and wound healing, but also in cancer development and progression. Furthermore, TGF-β1 has a dual role in cancer, as a tumor suppressor in earlier stages and tumor promoter in later stages [11]. TGF-β1 signaling falls into either the canonical Smad-dependent pathway or non-canonical Smad-independent pathway. The Smad-independent pathway includes the phosphoinositol-3 kinase (PI3K), mitogen-activated protein kinase (MAPK), and small guanosine triphosphatase (GTPase) pathways. These pathways are most often implicated in tumor cell motility and migration [12].

Gastric cancer is characterized by tumor heterogeneity. The Lauren classification is a well-known histologic classification system used in gastric cancer [13]. According to the Lauren classification, gastric cancer is categorized into intestinal (i-GC), diffuse (d-GC), or mixed types. Typically, i-GC tumors form gastric gland-like structures, while d-GCs do not. In addition to morphologic differences, these two types are known to have different epidemiologic, clinical, and molecular manifestations as well [14].

Our study aimed to investigate the expression pattern of TGF-β1 pathway-related molecules, including Smad-dependent and Smad-independent factors, in gastric cancers of different Lauren’s classifications.

## Methods

### Patients and samples

A series of 774 gastric cancer patients, who have undergone curative gastrectomy at Yonsei University Medical University Hospital (Seoul, Korea) from May 2005 to December 2005, were enrolled in this study. Patients who had a history of concurrent tumor, neoadjuvant chemotherapy or radiotherapy, preoperatively or intraoperatively detected metastasis, unavailable Lauren’s data, and follow-up loss were excluded from this study. The clinicopathologic data of the remaining 365 patients who satisfied the inclusion criteria were analyzed to investigate the clinicopathological differences between i-GC and d-GC. Patients were followed up clinically for at least 5 years after surgery, except in mortality cases. The follow-up time ranged from 3 to 69 months, with an average follow-up time of 55 months. We performed an analysis of mRNA microarray in the cases of 158 tissues among 365 cases as a pilot study. We observed that increased expression of TGF- β pathway gene modules were associated with unfavorable survival from that analysis (data not shown). Based on the results, further experiment of immunohistochemical study were planned to validate the clinical meaning of TGF- β signaling in gastric cancer subtypes. For more precise analysis, we decided to match the cases with considering the underlying clinic-pathological characteristics between the two subtypes of gastric cancer which might affect the interpretation of our results. A total of 68 tissues among 158 cases, therefore, were selected after some cases were dropped out because of the problems of tissue availability and readability. Written informed consent was obtained from all patients and the study was approved by the Institutional Review Board of Severance Hospital, Yonsei University (4-2012-0427).

### Immunohistochemistry

Paraffin-embedded sections (4 μm thick) were de-paraffinized with xylene and rehydrated in decreasing concentrations of ethanol. Sections were then incubated with 3 % H_2_O_2_ for 30 min at room temperature. The slides were immersed in 0.01 M citrate buffer (pH 6.0) for 10 min for antigen retrieval and then immersed in phosphate-buffered saline (PBS) containing 15 % goat serum. The primary antibodies were rabbit anti-human TGF-β1 polyclonal antibody (1:100; Santa Cruz, USA), mouse anti-human Smad4 monoclonal antibody (1:100; Santa Cruz, USA), mouse anti-human TGF-β receptor II (TβRII) monoclonal antibody (1:100; Santa Cruz, USA), rabbit anti-human phosphorylated (p)-Akt monoclonal antibody (1:100; Abcam, UK), rabbit anti-human p-extracellular signal-regulated kinase (ERK)1/2 polyclonal antibody (1:100; Santa Cruz, USA) and rabbit anti-human TGF-activated kinase 1 (TAK1) polyclonal antibody (1:100; Abcam, UK). After rinsing with PBS, secondary antibodies were added (goat anti-rabbit polymerized horse radish peroxidase [HRP]-labeled secondary antibody or goat anti-mouse IgG secondary antibody; Envision kit, DAKO, Denmark), and the slides placed in a thermostatic water bath at 37 °C for 30 min. After rinsing with PBS, the samples were counterstained using hematoxylin.

### Evaluation of results of immunohistochemical staining

**Fig. 1:**
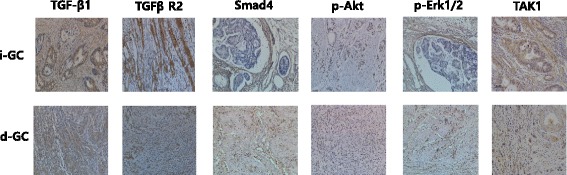
Immunohistochemical staining of TGF-β1 signaling molecules. TGFβR2, TGF receptor II; TAK1, TGF associated kinase 1; i-GC, intestinal type gastric cancer; d-GC, diffuse type gastric cancer. All cases were positively stained with each TGF-β1 signaling molecules. TGF-β1, TβR2 and TAK1 were stained in cell membrane and cytoplasm, while Smad4, p-Erk1/2 and p-Akt were stained in nucleus

Tissue samples were scored independently by two researchers (H.K. and K.H.P), who were blinded to the clinical data. Staining results for TGF-β1, TβR2, Smad4, TAK1, p-ERK1/2, and p-Akt were classified by estimating the percentage of epithelial cells exhibiting specific immunoreactivity: negative (no immunoreactivity); weak (0 to 33 % positive cells); moderate (33 to 67 % positive cells); and strong (>67 % positive cells) (Fig. 1). Only samples exhibiting moderate and strong immunoreactivity were considered positive. We used following cells as internal positive controls: inflammatory cells for TGF-β1 and p-Erk1/2; endothelial cells for TβR2 and Smad4; Myocytes for p-Akt1. For TAK1, it was difficult to find internal positive control (Additional file 1: Figure S1).

### Statistical analysis

Statistical analyses were performed using the SPSS statistical software program for Windows version 21 (SPSS Inc., Chicago, IL, USA). The association between variables was tested using Pearson’s χ^2^or Fisher’s exact tests for categorical variables. The survival data were analyzed using the Kaplan-Meier method and the log-rank test was used for assessing differences between groups. Two-sided values of *P* <0.05 were considered to indicate statistically significant differences.

## Results

### Differences in clinicopathologic features according to Lauren’s classification

The clinicopathologic data for patient groups sorted based on Lauren’s classification are summarized in Tables 1 and 2. Patients with d-GC were significantly younger (*P* <0.001) and more likely to be female (*P* <0.001). A larger proportion of the d-GC patients underwent total gastrectomy with more upper tumor location (*P* = 0.001) than i-GC patients. Patients with d-GC also had an increased propensity for recurrent disease (*P* = 0.028), in particular, peritoneal recurrence (*P* = 0.008). The overall survival rate of d-GC patients showed an unfavorable trend compared to that of i-GC patients (*P* = 0.072).

**Table 1:** Differences in demographic features of gastric cancer patients according to Lauren’s classification

		Intestinal-type	Diffuse-type	*P*-value
		(*n* = 204)	(*n* = 161)	
Age	<60 years	86	113	<0.001*
	>60 years	118	47	
Sex	Male	163	99	<0.001*
	Female	41	62	
Extent of resection	TG	30	47	0.001*
	STG	174	114	
Location	Fundus	16	48	0.001*
	Body	67	79	
	Antrum	117	63	
Recurrence	(−)	172	122	0.028*
	(+)	32	39	
Recurrence site	Hematogenous	11	4	0.008*
	Peritoneal	11	27	
	Locoregional	3	4	
	Mixed	7	3	
Survival	Alive	175	126	0.072
	Dead	29	35	

*P <0.05, calculated using; *N.S.* not significant, *TG* total gastrectomy, *STG* subtotal gastrectomy

**Table 2:** Differences in pathological characteristics according to Lauren’s classification

		Intestinal-type	Diffuse-type	*P*-value
		(*n* = 204)	(*n* = 161)	
Size	<30 mm	134	88	0.021*
	>30 mm	70	73	
Differentiation	Differentiated	188	4	<0.001*
	Poorly differentiated	16	157	
LVI	(−)	140	97	0.099
	(+)	64	64	
T stage	T1	126	70	<0.001*
	T2	17	13	
	T3	33	19	
	T4	28	59	
N stage	N0	146	92	0.004*
	N1	22	14	
	N2	11	17	
	N3	25	38	

*P <0.05, calculated using; *N.S.* not significant, *LVI* lymphovascular invasion

Tumors in patients with d-GC were larger (*P* = 0.021), more undifferentiated (*P* <0.001), and of higher T- (*P* <0.001) and N-stages (*P* = 0.004) than those in i-GC patients. Lymphovascular invasion (LVI) was not different between the two patient groups (*P* = 0.099).

After selecting stage matched gastric cancer patients according to Lauren’s classification (Table 3), none of the parameters between i-GC and d-GC patients showed statistically significant differences, with the exception of patient age (younger in d-GC; *P* <0.01).

**Table 3:** Clinicopathologic characteristics of 68 randomly selected gastric cancer patients whose tumors were utilized for immunohistochemical analysis

		Intestinal-type	Diffuse-type	*P*-value
		(*n* = 33)	(*n* = 35)	
Age	<60 years	10	25	<0.01
	>60 years	23	10	
Sex	Male	24	25	NS
	Female	9	10	
Depth of invasion	T1	9	10	NS
	≥T2	24	25	
LN metastasis	Negative	17	20	NS
	Positive	16	15	
TNM stage*	I	8	11	NS
	II	14	16	
	III	11	8	

* 7^th^ ed., N.S. not significant

### Expression of TGF-β1 signaling molecules

Positive expression rate of TGF-β1 signaling molecules in i-GC and d-GC, respectively, was as follows; TGF-β1 (61 and 34 %), TβR2 (100 and 66 %), Smad4 (26 and 27 %), p-ERK1/2 (82 and 37 %), p-Akt (72 and 69 %), and TAK1 (87 and 83 %) (Fig. 1; Expression data is summarized in Table 4).

**Table 4:** Expression of TGF-β signaling molecules according to Lauren’s classification

		Intestinal-type	Diffuse-type	*P*-value
		(*n* = 33)	(*n* = 35)	
TGF-β1	Low	13	23	0.050
	High	20 (61 %)	12 (34 %)	
TβR2	Low	0	12	<0.001
	High	33 (100 %)	23 (66 %)	
Smad4	Low	23	25	0.588
	High	8 (26 %)	9 (27 %)	
p-ERK1/2	Low	3	12	0.008
	High	14 (82 %)	7 (37 %)	
p-Akt	Low	9	11	0.796
	High	23 (72 %)	24 (69 %)	
TAK1	Low	4	6	0.758
	High	27 (87 %)	29 (84 %)	

*TGF-β1* transforming growth factor-β1, *T βR2* TGF-β receptor 2, *p-ERK* phosphorylated extracellular signal-regulated kinase, *TAK1* TGF-activated kinase1

TGF-β1 and TβR2 were activated in both gastric cancer histologic subtypes. However, all the TGF-β1 signaling pathway molecules were expressed at higher levels in i-GC than in d-GC tumors (*P* = 0.05, *P* < 0.001 in all). The co-expression rate of TGF-β ligand and receptor was much higher in i-GC (20/33, 64.5 %) than in d-GC tumors (11/37, 35.5 %; *P* = 0.015). The expression of Smad4, a representative molecule of the Smad-dependent pathway, was decreased in both subtypes, albeit without statistically significant difference between the subtypes (*P* = 0.588). Among factors involved in the Smad-independent pathway, p-Akt and TAK1 were over-expressed, without a statistically significant difference between i-GC and d-GC (*P* = 0.796 and *P* = 0.742, respectively). Of note, the expression of p-ERK1/2 was higher in i-GC than in d-GC (*P* = 0.008).

**Fig. 2:**
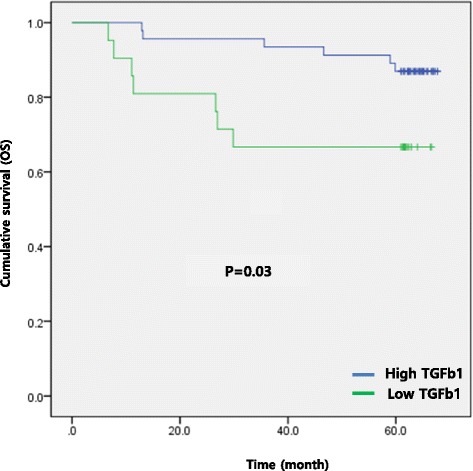
Kaplan-Meier analysis of overall survival between high TGF-β1 signal and low TGF-β1 signal. Patients group with high TGF-β1 showed poorer overall survival (50.2 ± 5.2 months vs. 63.8 ± 1.8 months, *p* = 0.03)

Further, the increased TGF-β1 signaling pathway was significantly correlated with poor overall survival in gastric cancer patients (50.2 ± 5.2 months vs. 63.8 ± 1.8 months, *P* = 0.03) (Fig. 2).

## Discussion

In this study, we identified significant clinicopathologic differences between i-GC and d-GC. Our study could support the possibility of the conversion of TGF- β1 signaling from a Smad-dependent to a Smad-independent pathway in case of malignant status, a hypothesis suggested in one previous study [15]. In addition, we propose that gastric cancer subtypes may utilize a different Smad-independent signaling pathway. Our data suggest that i-GC might depend more on the p-ERK1/2 pathway than d-GC. It can be supported by the previous study [16] which reported that p-ERK1/2 pathway is associated with *H. pylori* infection- well known proven cause of i-GC [17].

TGF-β1 has the potential to function as a tumor suppressor (via its effects on proliferation, replication potential, and apoptosis), or as a tumor promoter (via its effects on migration, invasion, angiogenesis, and the immune system) [11]. Based on animal models and in vitro studies, Elliot and Blobe [18] proposed a hypothesis, in which during early tumorigenesis, TGF-β1-mediated tumor suppressor activity functions through a Smad-dependent pathway, while tumor promoter activity acts through a Smad-independent pathway. However, no complete clinical study included empirical data supporting this hypothesis, except that of Kim et al. [15]. Their analysis of a 332-tissue microarray, which was performed along the normal epithelium-atrophic gastritis-dysplasia-carcinoma sequence, showed that TGF-β1 and TβR1 expression continually increased along this sequence, while Smad 2/3 and Smad4 decreased as carcinoma progressed. Their study, however, was limited because they did not evaluate the expression of Smad-independent signaling molecules. In our present study, we evaluated two TGF-β1 pathways in gastric cancers by assessing the expression of Smad -dependent and Smad -independent signaling molecules, although we did not compare the expression of TGF-β1 signaling molecules between tumor and normal tissue. When considering our data and the study of Kim et al. [15], we suggest a model for the role of TGF-β in gastric carcinogenesis, whereby TGF-β1 signaling changes from the tumor-suppressive Smad-dependent pathway to a tumor-activating Smad-independent pathway as the cancer progresses, irrespective of histologic subtypes of gastric cancer.

The main sources of TGF-β1 are stromal cells, such as fibroblasts, lymphocytes, and macrophages [12]. Therefore, the expression of TGF-β1 within tumors is higher in d-GC than in i-GC, largely because d-GC has more stromal components [5]. However, our study, in which we assessed the immunoreactivity of only cancer cells not stroma, revealed another interesting aspect of TGF-β signaling in gastric cancer; the expression of ligand and receptor of TGF-β1 signaling was increased in gastric cancer tissue, which suggests a potential autocrine loop in gastric cancer. Interestingly, in our data, co-expression of both ligand and receptor was higher in i-GC than in d-GC. It is thus conceivable that a paracrine effect of TGF-β1 signaling is dominant in d-GC, in which cancerous stromal cells are abundant, while an autocrine function of TGF-β1 might play an important role in i-GC. We will address this hypothesis in our future studies.

We note the limitations of our current study; our sample of gastric cancer tissues was relatively small and had a possibility of selection bias. In addition, we did not compare the expression of TGF-β1 signaling molecules in cancer tissues with that in matched normal tissues.

Therapeutic strategies targeting TGF-β1 signaling in cancer treatment is a burgeoning field of research [19]. The current results can augment our understanding of the role of TGF-β1 signaling in distinct histologic subtypes of gastric cancer.

## Conclusions

The clinicopathological characteristics vary in different histologic gastric cancer subtypes. Although TGF-β1 signaling in gastric cancer cells appears hyper-activated in i-GC compared to d-GC, the Smad-dependent pathway seems down-regulated while the Smad-independent pathway seems up-regulated in both histologic subtypes. Of note, the expression of p-ERK1/2, a Smad-independent pathway factor, was significantly increased in i-GC.

## Additional file

Additional file 1: Figure S1. Following cells were used as internal positive controls: inflammatory cells for TGF-β1 and p-Erk1/2; endothelial cells for TβR2 and Smad4; Myocytes for p-Akt1. For TAK1, it was difficult to find internal positive control. (PPTX 13592 kb)

## Abbreviations

i-GC: intestinal type gastric cancer
d-GC: diffuse type gastric cancer
LVI: lymphovascular invasion
TAK1: TGF associated kinase 1
