# Erratum to: Differences in TGF-β1 signaling and clinicopathologic characteristics of histologic subtypes of gastric cancer

**DOI:** 10.1186/s12885-016-2140-5

**Published:** 2016-02-15

**Authors:** Kyung Ho Pak, Dong Hoon Kim, Hyunki Kim, Do Hyung Lee, Jae-Ho Cheong

**Affiliations:** Department of Surgery, Hallym University Medical Center, Hwasung, Korea; Department of Pathology, Hallym University Medical Center, Hwasung, Korea; Department of Medicine, Yonsei University Graduate School, Seoul, Korea; Department of Pathology, Yonsei University College of Medicine, Seoul, Korea; Depatment of Surgery, Yonsei University College of Medicine, 50-1 Yonsei-ro, Seoul, Seodaemun-gu 120-752 Korea; Department of Biochemistry & Molecular Biology, Yonsei University College of Medicine, Seoul, Korea; Brain Korea 21 PLUS Project for Medical Science, Yonsei University College of Medicine, Seoul, Korea; Open NBI Convergence Technology Research Laboratory, Yonsei University College of Medicine, Seoul, Korea

## Erratum

Unfortunately, the original version of this article [1] contained an error in Fig. 2. Within this figure, the annotations for “High TGFb1” and “Low TGFb1” were the wrong way around. This has been corrected in the original article and is also included correctly below.

**Fig. 2:**
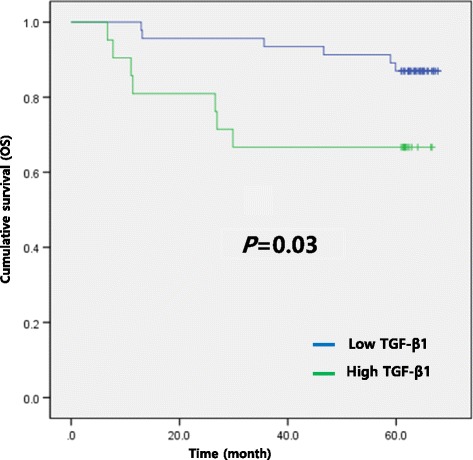
ᅟ
