# Supplementary figures and images for: Differences in TGF-β1 signaling and clinicopathologic characteristics of histologic subtypes of gastric cancer
Source: BMC Cancer. 2016 Feb 4;16:60. doi: 10.1186/s12885-016-2091-x (PMC4743329; doi:10.1186/s12885-016-2091-x)

## Slide 1
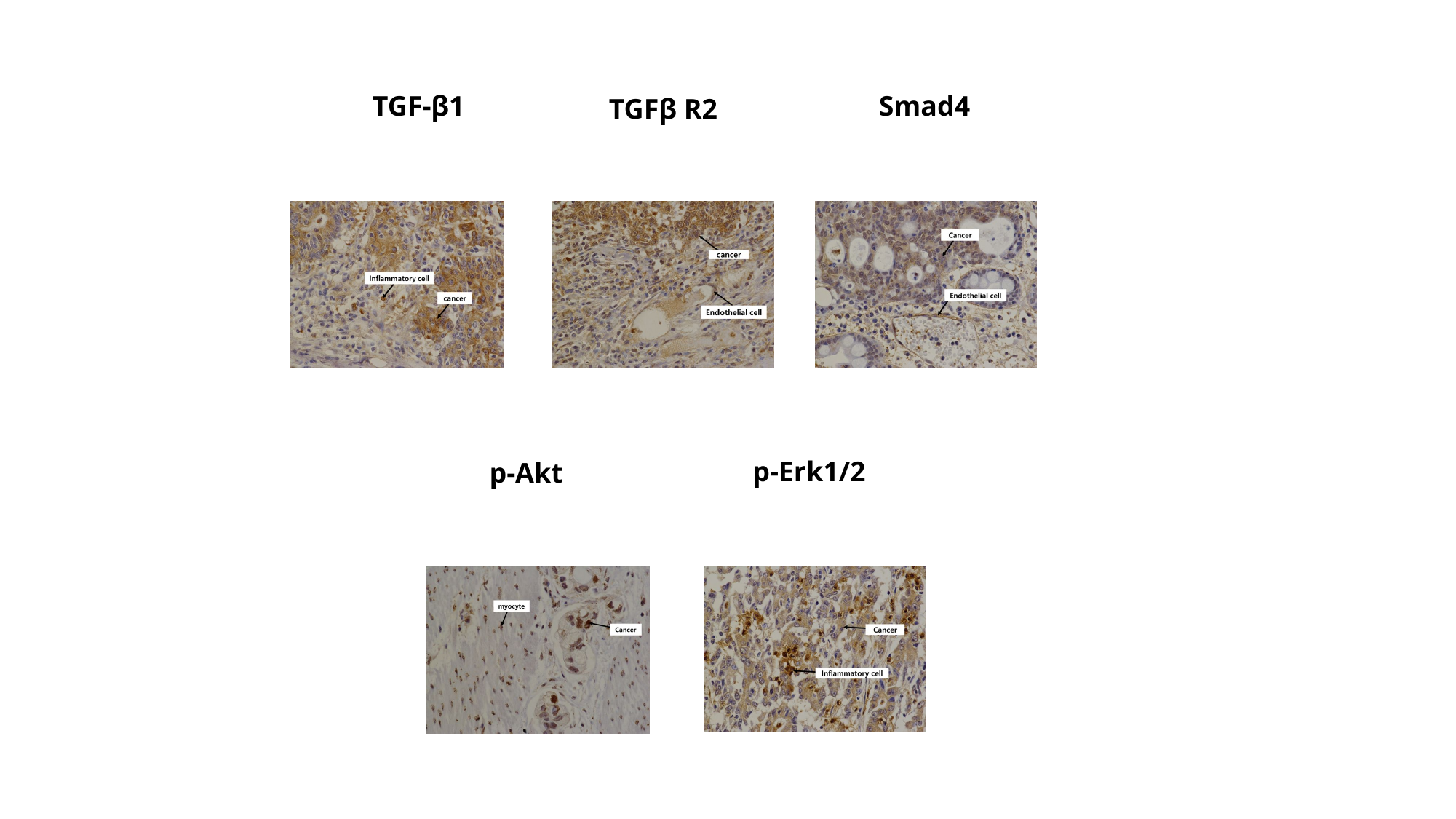

TGF-β1
Smad4
TGFβ R2
p-Erk1/2
p-Akt

Supplement: Additional file 1: Figure S1. — Following cells were used as internal positive controls: inflammatory cells for TGF-β1 and p-Erk1/2; endothelial cells for TβR2 and Smad4; Myocytes for p-Akt1. For TAK1, it was difficult to find internal positive control. (PPTX 13592 kb) [file 12885_2016_2091_MOESM1_ESM.pptx]
